# A Finite-Difference Approach for Plasma Microwave Imaging Profilometry

**DOI:** 10.3390/jimaging5080070

**Published:** 2019-08-12

**Authors:** Loreto Di Donato, David Mascali, Andrea F. Morabito, Gino Sorbello

**Affiliations:** 1 Department of Electrical, Electronics and Computer Engineering (DIEEI), University of Catania, viale A. Doria 6, 95126 Catania, Italy; 2 Laboratori Nazionali del Sud, National Institute of Nuclear Physics (INFN), Via Santa Sofia 62, 95125 Catania, Italy; 3 Department of Information Engineering, Infrastructures and Sustainable Energy (DIIES), University “Mediterranea” of Reggio Calabria, via Graziella, Loc. Feo di Vito, 89124 Reggio di Calabria, Italy

**Keywords:** microwave plasma diagnostics, electromagnetic inverse scattering, microwave imaging profilometry, finite-difference methods

## Abstract

Plasma diagnostics is a topic of great interest in the physics and engineering community because the monitoring of plasma parameters plays a fundamental role in the development and optimization of plasma reactors. Towards this aim, microwave diagnostics, such as reflectometric, interferometric, and polarimetric techniques, can represent effective means. Besides the above, microwave imaging profilometry (MIP) may allow the obtaining of tomographic, i.e., volumetric, information of plasma that could overcome some intrinsic limitations of the standard non-invasive diagnostic approaches. However, pursuing MIP is not an easy task due to plasma’s electromagnetic features, which strongly depend on the working frequency, angle of incidence, polarization, etc., as well as on the need for making diagnostics in both large (meter-sized) and small (centimeter-sized) reactors. Furthermore, these latter represent extremely harsh environments, wherein different systems and equipment need to coexist to guarantee their functionality. Specifically, MIP entails solution of an inverse scattering problem, which is non-linear and ill-posed, and, in addition, in the one-dimensional case, is also severely limited in terms of achievable reconstruction accuracy and resolution. In this contribution, we address microwave inverse profiling of plasma assuming a high-frequency probing regime when magnetically confined plasma can be approximated as both an isotropic and weak penetrable medium. To this aim, we adopt a finite-difference frequency-domain (FDFD) formulation which allows dealing with non-homogeneous backgrounds introduced by unavoidable presence of plasma reactors.

## 1. Introduction

Plasma diagnostics using active microwave techniques is one of the most promising tools to optimize the production of plasma in both large and compact reactors because of its non-invasive nature and relatively modest access requirements [1]. Among the others, microwave imaging reflectometry (MIR), interferometric and polarimetric techniques are currently under investigation to extract plasma proprieties useful to model and optimize the heating process. Such information is crucial to study and improve plasma generation based on Electron Cyclotron Resonance [2] as well as innovative heating schemes such as the ones employing Electrostatic Bernstein Waves in overdense plasma [3]. Unfortunately, the diagnostic approaches mentioned above show some limitations. As an example, MIR, a multifrequency radar-like technique, mainly used in large reactors, such as tokamak and stellarator, is commonly used to infer electronic density distribution and fluctuation, but suffers from the impossibility of performing diagnostics of non-increasing plasma profiles since inner reflecting cutoff layers are “hidden” from the outer ones [4]. On the other hand, line-integrated measurements, such as those actually provided by interferometric and polarimetric techniques, although employable on compact reactors such as the electron cyclotron ion source (ECRIS) [5], cannot give local information about electron density, but only a line-of-sight integrated value.

For these reasons, it is worth devoting interest towards microwave imaging tomographic approaches, such as those actually used in medical and subsurface microwave diagnostics [6], which are in principle able to “locally” monitor plasma uniformity and instabilities.

In a recent paper, we investigated the possibility of obtaining plasma imaging profilometry (MIP) by means of electromagnetic inverse scattering techniques requiring only measurements of the reflection coefficient in a quite large frequency band. In particular, adopting a high-frequency probing regime (i.e., probing frequencies much larger than the cyclotron frequency), i.e., when the plasma can be assumed to be both isotropic and weak scatterer, linear imaging recovery techniques are able to achieve quantitative characterization of plasma profiles [7]. The results have shown that if the recovery process is properly formulated as a sparse optimization problem, compressive sensing (CS)-based strategies are able to achieve nearly optimal results. However, free-space homogeneous background has been considered, which is an assumption suitable only if multiple reflections introduced by the metallic environment (reactor walls) are neglected or, in any case, filtered out.

With the aim to get a further step in the analysis of such a problem, in this paper we introduce a frequency-domain finite-difference (FDFD) formulation for the solution of the inverse scattering problem in order to take easily into account the effect of non-homogeneous backgrounds related to the metallic environment of the plasma chamber.

On the other hand, besides the modeling aspects, solution of the inverse scattering problem in presence of a metallic surface, or, in general, non-homogeneous background, can bring some advantages due to the possibility to achieve enhanced resolution in the reconstruction process [8,9]. Roughly speaking, this enhancement can be explained due to the multiple reflected waves which can bring additional information conveyed back by the recovery process. Interestingly, this may be of fundamental importance in the solution of the one-dimensional inverse scattering problem (also known as inverse profiling) wherein the number and kind of retrievable information is severely limited [10].

The paper is structured as follows. In Section 2, the adopted mathematical formulation for the solution of the forward and inverse scattering problem is described. In Section 3 the microwave imaging profilometry for inhomogeneous plasma slabs recovery is formulated. Finally, in Section 4, the developed approach is validated against simulated data dealing with homogeneous and non-homogeneous backgrounds as well as reflection-only and reflection-transmission measurement configuration. Conclusions follow.

## 2. Mathematical Formulation

Let us assume a bounded, simply connected, domain *D* located in an non-homogeneous background medium, wherein some electromagnetic primary sources $\underline{j\;}{\;}_{0}$ are located in a region characterized by relative permittivity and permeability distribution $\mu_{r}\left(\underline{r\;}{\;}^{\prime }\right)$ and $\epsilon_{r}\left(\underline{r\;}{\;}^{\prime }\right)$, $\underline{r\;}{\;}^{\prime }$ spanning the space *D*. After normalizing the electric field, magnetic field and the primary sources according to $\tilde{\underline{e\;}\;}=\sqrt{\frac{\epsilon_{0}}{\mu_{0}}}\underline{e\;}\;$, $\tilde{\underline{h\;}\;}=\underline{h\;}\;$ and ${\tilde{\underline{j\;}\;}}_{0}=c_{0}\underline{j\;}{\;}_{0}$, with $c_{0}=\frac{1}{\sqrt{\epsilon_{0}\mu_{0}}}$ the speed of light, Maxwell equations read:

(1) $$\begin{array}{c} c_{0}\underline{\nabla \;}\;\times \tilde{\underline{e\;}\;}=-j\omega \mu_{r}\left(\underline{r\;}{\;}^{\prime }\right)\tilde{\underline{h\;}\;}\\ c_{0}\underline{\nabla \;}\;\times \tilde{\underline{h\;}\;}=j\omega \epsilon_{r}\left(\underline{r\;}{\;}^{\prime }\right)\tilde{\underline{e\;}\;}+{\tilde{\underline{j\;}\;}}_{0} \end{array}\;\;$$

where ω is the angular frequency and the time factor exp{jωt} is assumed and dropped for the time-harmonic fields. Normalized electric and magnetic fields, $\tilde{\underline{e\;}\;}$ and $\tilde{\underline{h\;}\;}$, are of the same order of magnitude and this is an advantage in formulating the perfectly matched layer (PML) [11]. If, for the sake of a simpler notation, we drop the tilde $\tilde{}$ symbol and the $c_{0}$ is included in the $\underline{\nabla \;}\;\times$ definition, we can write:

(2) $$\begin{array}{c} \underline{\tilde{\nabla }\;}\;\times \underline{e\;}\;=-j\omega \mu_{r}\left(\underline{r\;}{\;}^{\prime }\right)\underline{h\;}\;\\ \underline{\tilde{\nabla }\;}\;\times \underline{h\;}\;=j\omega \epsilon_{r}\left(\underline{r\;}{\;}^{\prime }\right)\underline{e\;}\;+\underline{j\;}{\;}_{0} \end{array}\;\;$$

In the above formulation, to satisfy different boundary conditions, a lossy PML [12] can be introduced to truncate the computational domain, as commonly done in numerical solver. By choosing proper PML parameters, various boundary conditions can seamlessly introducing ranging from a perfect absorbing boundary condition to a PEC (or PMC) boundary condition, the latter case when no PML is used [11].

The background field (i.e., the field without the unknown profile) defined as a plane wave propagating in a non-homogeneous, eventually lossy, background with permittivity and permeability, respectively, given by:

$$\epsilon_{b}\left(\underline{r\;}{\;}^{\prime }\right)=\epsilon_{b}^{\prime }\left(\underline{r\;}{\;}^{\prime }\right)-j\epsilon_{b}^{\prime \prime }\left(\underline{r\;}{\;}^{\prime }\right)=\epsilon_{b}^{\prime }\left(\underline{r\;}{\;}^{\prime }\right)-j\frac{\sigma (\underline{r\;}{\;}^{\prime })}{\epsilon_{0}\omega }$$

$$\mu_{b}\left(\underline{r\;}{\;}^{\prime }\right)=\mu_{b}^{\prime }\left(\underline{r\;}{\;}^{\prime }\right)-j\mu_{b}^{\prime \prime }\left(\underline{r\;}{\;}^{\prime }\right)$$

satisfies the following equations:

(3) $$\begin{array}{c} \underline{\tilde{\nabla }\;}\;\times \underline{e\;}{\;}_{bck}=-j\omega \mu_{b}\left(\underline{r\;}{\;}^{\prime }\right)\underline{h\;}{\;}_{bck}\\ \underline{\tilde{\nabla }\;}\;\times \underline{h\;}{\;}_{bck}=j\omega \epsilon_{b}\left(\underline{r\;}{\;}^{\prime }\right)\underline{e\;}{\;}_{bck}+\underline{j\;}{\;}_{0} \end{array}\;$$

To formulate the scattering problem, we can add and subtract the quantities $j\omega \epsilon_{b}\left(\underline{r\;}{\;}^{\prime }\right)\underline{e\;}\;$ and $j\omega \mu_{b}\left(\underline{r\;}{\;}^{\prime }\right)\underline{h\;}\;$ at the right hand side member of Equation (2) to obtain:

(4) $$\begin{array}{c} \underline{\tilde{\nabla }\;}\;\times \underline{e\;}\;=-j\omega \mu_{b}\left(\underline{r\;}{\;}^{\prime }\right)\underline{h\;}\;-j\omega \left[\mu_{r}\left(\underline{r\;}{\;}^{\prime }\right)-\mu_{b}\left(\underline{r\;}{\;}^{\prime }\right)\right]\underline{h\;}\;\\ \underline{\tilde{\nabla }\;}\;\times \underline{h\;}\;=j\omega \epsilon_{b}\underline{e\;}\;+j\omega \left[\epsilon_{r}\left(\underline{r\;}{\;}^{\prime }\right)-\epsilon_{b}\left(\underline{r\;}{\;}^{\prime }\right)\right]\underline{e\;}\;+\underline{j\;}{\;}_{0} \end{array}\;\;$$

and, finally, subtracting (3) to (4), the equations governing the scattered fields, defined as $\underline{e\;}{\;}_{sct}=\underline{e\;}\;-\underline{e\;}{\;}_{bck}$ and $\underline{h\;}{\;}_{sct}=\underline{h\;}\;-\underline{h\;}{\;}_{bck}$ , are given by:

(5) $$\begin{array}{c} \underline{\tilde{\nabla }\;}\;\times \underline{e\;}{\;}_{sct}=-j\omega \mu_{b}\left(\underline{r\;}{\;}^{\prime }\right)\underline{h\;}{\;}_{sct}-j\omega \mu_{b}\left(\underline{r\;}{\;}^{\prime }\right)\left[\frac{\mu_{r}\left(\underline{r\;}{\;}^{\prime }\right)-\mu_{b}\left(\underline{r\;}{\;}^{\prime }\right)}{\mu_{b}\left(\underline{r\;}{\;}^{\prime }\right)}\right]\underline{h\;}\;\\ \underline{\tilde{\nabla }\;}\;\times \underline{h\;}{\;}_{sct}=j\omega \epsilon_{b}\left(\underline{r\;}{\;}^{\prime }\right)\underline{e\;}{\;}_{sct}+j\omega \epsilon_{b}\left(\underline{r\;}{\;}^{\prime }\right)\left[\frac{\epsilon_{r}\left(\underline{r\;}{\;}^{\prime }\right)-\epsilon_{b}\left(\underline{r\;}{\;}^{\prime }\right)}{\epsilon_{b}\left(\underline{r\;}{\;}^{\prime }\right)}\right]\underline{e\;}\; \end{array}\;$$

wherein the quantities under the square brackets can be easily recognized as the contrast functions:

$$\chi_{\mu }\left(\omega ,\underline{r\;}{\;}^{\prime }\right)=\frac{\mu_{r}\left(\omega ,\underline{r\;}{\;}^{\prime }\right)-\mu_{b}\left(\omega ,\underline{r\;}{\;}^{\prime }\right)}{\mu_{b}\left(\omega ,\underline{r\;}{\;}^{\prime }\right)},$$

$$\chi_{\epsilon }\left(\omega ,\underline{r\;}{\;}^{\prime }\right)=\frac{\epsilon_{r}\left(\omega ,\underline{r\;}{\;}^{\prime }\right)-\epsilon_{b}\left(\omega ,\underline{r\;}{\;}^{\prime }\right)}{\epsilon_{b}\left(\omega ,\underline{r\;}{\;}^{\prime }\right)},$$

which relate the dielectric and magnetic properties of the anomalies, i.e., scattering objects, to those of the embedding background medium at each frequency.

Adopting a dual grid [13,14] and a proper discretization, in operator notation, the above equations can be rewritten as:

(6) $$\left[\begin{array}{cc} C & j\omega M_{b}\\ -j\omega E_{b} & C \end{array}\right]\left[\begin{array}{c} \underline{e\;}{\;}_{sct}^{\prime }\\ \underline{h\;}{\;}_{sct}^{\prime } \end{array}\right]=\left[\begin{array}{cc} 0 & -j\omega \mu_{b}\chi_{\mu }\\ j\omega \epsilon_{b}\chi_{\epsilon } & 0 \end{array}\right]\left[\begin{array}{c} \underline{e\;}{\;}^{\prime }\\ \underline{h\;}{\;}^{\prime } \end{array}\right],\;\;$$

wherein ${\left(\right)}^{\prime }$ is used to indicate the field integrated over the appropriate line element of the primal or dual mesh ($\underline{e\;}{\;}^{\prime }\approx \underline{e\;}\;\bar{\Delta L}$, $\underline{h\;}{\;}^{\prime }\approx \underline{h\;}\;\tilde{\Delta L}$). Moreover, the matrix operator C known in the literature as incidence matrix (see for example section §7.3.3 and Equation (7.1) of Tonti’s book [13] or [15]) denotes the proper derivative operations and is a sparse matrix. Moreover, $M_{b}$ and $E_{b}$ are matrices accounting for the magnetic and dielectric non-homogeneous background properties, respectively, wherein $E_{b}\approx \epsilon_{b}\frac{\tilde{\Delta S}}{\bar{\Delta L}}$ and $M_{b}\approx \mu_{b}\frac{\bar{\Delta S}}{\tilde{\Delta L}},$ with $\bar{\Delta L}$ and $\bar{\Delta S}$ refer to lines and surfaces of the primal mesh, and $\tilde{\Delta L}$ and $\tilde{\Delta S}$ refer to the dual mesh [13]. In the remaining of the paper for the sake of a simpler notation we drop the ${}^{\prime }$ symbol.

The formal solution of the linear system (6) can be rewritten as:

(7) $$\left[\begin{array}{c} \underline{e\;}{\;}_{sct}\\ \underline{h\;}{\;}_{sct} \end{array}\right]=H_{b}^{-1}\left(\left[\begin{array}{cc} 0 & -j\omega \mu_{b}\chi_{\mu }\\ j\omega \epsilon_{b}\chi_{\epsilon } & 0 \end{array}\right]\left[\begin{array}{c} \underline{e\;}\;\\ \underline{h\;}\; \end{array}\right]\right).\;\;$$

The above equation is the key equation for the inverse scattering problem adopting a finite-difference formulation. As a matter of fact, the measured scattered fields, i.e., the scattered field recorded on the data domain *S* (for the case at hand, the same positions $z=z_{S}$ at different frequencies), can be represented by the following operator notations:

(8) $$\left[\begin{array}{c} \underline{e\;}{\;}_{sct}\left(z_{S},\omega_{n}\right)\\ \underline{h\;}{\;}_{sct}\left(z_{S},\omega_{n}\right) \end{array}\right]=M_{S}\left\{H_{b}^{-1}\left(\left[\begin{array}{cc} 0 & -j\omega \mu_{b}\chi_{\mu }\\ j\omega \epsilon_{b}\chi_{\epsilon } & 0 \end{array}\right]\left[\begin{array}{c} \underline{e\;}\;\\ \underline{h\;}\; \end{array}\right]\right)\right\},\;\;$$

where $M_{S}$ is an operator that extracts the field values at the observation point $z=z_{S}$ at different frequencies. Substituting ${\left[\underline{e\;}{\;}_{sct}\;,\;\underline{h\;}{\;}_{sct}\right]}^{T}={\left[\underline{e\;}\;\;,\;\underline{h\;}\;\right]}^{T}-{\left[\underline{e\;}{\;}_{bck}\;,\;\underline{h\;}{\;}_{bck}\right]}^{T}$ into (7) , we obtain the domain (or object) equation:

(9) $$\left[\begin{array}{c} \underline{e\;}{\;}_{bck}\\ \underline{h\;}{\;}_{bck} \end{array}\right]=\left[\begin{array}{c} \underline{e\;}\;\\ \underline{h\;}\; \end{array}\right]-\left\{H_{b}^{-1}\left(\left[\begin{array}{cc} 0 & -j\mu_{b}\omega \chi_{\mu }\\ j\omega \epsilon_{b}\chi_{\epsilon } & 0 \end{array}\right]\left[\begin{array}{c} \underline{e\;}\;\\ \underline{h\;}\; \end{array}\right]\right)\right\},\;$$

that can be sampled with an operator, say it $M_{D}$, that extracts fields only in the imaging domain *D* at the discretization grid points $z_{D}$ [16], i.e., :

(10) $$\left[\begin{array}{c} \underline{e\;}{\;}_{bck}\left(z_{D},\omega_{n}\right)\\ \underline{h\;}{\;}_{bck}\left(z_{D},\omega_{n}\right) \end{array}\right]-\left[\begin{array}{c} \underline{e\;}\;\left(z_{D},\omega_{n}\right)\\ \underline{h\;}\;\left(z_{D},\omega_{n}\right) \end{array}\right]=M_{D}\left\{H_{b}^{-1}\left(\left[\begin{array}{cc} 0 & -j\omega \mu_{b}\chi_{\mu }\\ j\omega \epsilon_{b}\chi_{\epsilon } & 0 \end{array}\right]\left[\begin{array}{c} \underline{e\;}\;\\ \underline{h\;}\; \end{array}\right]\right)\right\}.\;$$

Equations (8)–(10) are the basic equations to solve the inverse scattering problem which is non-linear since both the contrast functions $\chi_{\epsilon }$ and $\chi_{\mu }$ and the field $\underline{e\;}\;$ and $\underline{h\;}\;$ are unknowns of the problem [17]. Moreover, the problem is ill-posed, too, and effective regularization strategies must be adopted for stable solutions against the presence of noise on data [18].

## 3. Microwave Imaging Profilometry

### 3.1. Linearized Approach for Plasma Slabs

**Figure 1:**
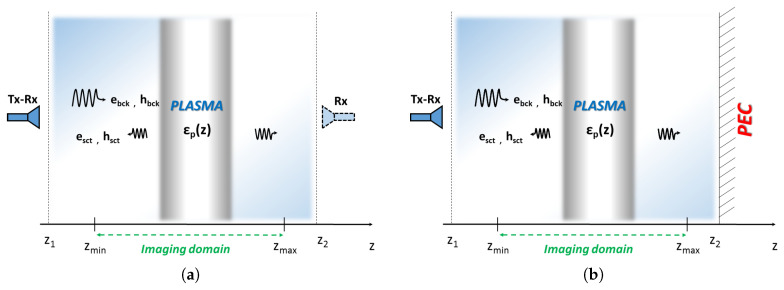
Sketch of the single view normal incidence measurement. The imaging domain extents in the range $\left[z_{min},z_{max}\right]$ and the scattered field data at different frequencies are gathered at a given observation points external to this range. (**a**) Homogeneous background: configuration with only an active (tx-rx) antenna (neglecting the rightmost dashed antenna) and an active (tx-rx) and a passive (rx) antenna (considering the rightmost dashed antenna). (**b**) Non-homogeneous background: configuration with an active antenna (tx-rx) with a PEC surface.

Let us consider a one-dimensional non-homogeneous slab extending in the range $\left[z_{1},z_{2}\right]$ embedded in a non-homogeneous background, see Figure 1a. Normal incidence plane waves are used to probe the slab at difference frequencies with phase reference equal to zero at $z=z^{\left(\mathrm{View}\right)}\left(z\geq z_{1}\right)$. Accordingly, the scattered field can be gathered at a given abscissa $z_{1}\leq z_{S}=z^{\left(\mathrm{Meas}\right)}\leq z_{min}$ (reflection measurement) or $z_{max}\leq z_{S}=z^{\left(\mathrm{Meas}\right)}\leq z_{2}$ (transmission measurement).

To retrieve nonmagnetic ($\mu_{r}\left(\underline{r\;}{\;}^{\prime }\right)=\mu_{b}$) dielectric profile of inhomogeneous isotropic plasma slabs, we consider a first order linearized approach under the Rytov approximation, instead of solving the exact optimization problems stated by Equations (8)–(10). Linearized approaches, such as the Born (BA) and Rytov (RA) approximations, can be used to recover small and extended (with respect to the wavelength) weak slabs, respectively, i.e., slabs showing low contrast ($\left|\right|\chi_{\epsilon }\left|\right|<<1$). In particular, the Rytov approximation is useful for scatterers far extended in terms of the probing wavelengths (anyway, RA reduces to BA when the scatterers become smaller and smaller with respect to the probing wavelengths [19]). In this respect, to fulfill model hypothesis, it is worth noticing that assuming a relatively high-frequency probing band (with respect to the cyclotron frequency) entails several positive fallouts. First, at probing frequencies much higher than the cyclotron frequency, the plasma can be assumed as an isotropic medium since the permittivity tensor becomes diagonal with the terms all equals each other [2,20]. Second, at probing frequencies such that $\omega^{2}>>\nu^{2}$ the plasma contrast becomes:

(11) $$\chi_{\epsilon }=-\frac{\omega_{p}^{2}}{\omega^{2}+\nu^{2}}-j\frac{\omega_{p}^{2}\nu }{\omega (\omega^{2}+\nu^{2})}\approx -\frac{\omega_{p}^{2}}{\omega^{2}}-j\frac{\omega_{p}^{2}\nu }{\omega^{3}},$$

wherein:

(12) $$\omega_{p}=\sqrt{\frac{n_{e}e^{2}}{m_{e}\epsilon_{0}}},$$

$n_{e}$, *e* and $m_{e}$ being the electron density, electron charge, and electron mass, respectively. Under the above approximations, considering the frequency difference (FD) formulation described in Section 2, the unknown total electric field can be approximated by the incident field according to the RA, to obtain the following linear equation:

(13) $$\underline{f\;}{\;}_{sct}=M_{S}\left\{{\tilde{R}}_{b}^{-1}\chi_{\epsilon }\underline{e\;}{\;}_{bck}\right\},$$

wherein $\underline{f\;}{\;}_{sct}=\underline{e\;}{\;}_{sct}/\underline{e\;}{\;}_{bck}$ and ${\tilde{R}}_{b}^{-1}$, replacing ${\tilde{H}}_{b}^{-1}$ unless unessential constant, takes into account the normalization by the incident field values at the measurement point $z^{\left(\mathrm{Meas}\right)}$ introduced by the Rytov approximation [19] and the frequency dependence of the (multifrequency) scattering operator. As a result, in order to solve for the plasma constitutive parameters $\omega_{p}^{2}$ and ν, the frequency dependent complex valued problem (13), can be recast in terms of frequency independent real valued problem as already exploited in [7], i.e.,:

(14) $$\left[\begin{array}{c} Re(\underline{f\;}{\;}_{sct})\\ Im(\underline{f\;}{\;}_{sct}) \end{array}\right]=\left[\begin{array}{cc} -\frac{1}{\omega^{2}}M_{S}Re\left({\tilde{R}}_{b}^{-1}\underline{e\;}{\;}_{bck}\right) & \;\;\;\;\frac{1}{\omega^{3}}M_{S}Im\left({\tilde{R}}_{b}^{-1}\underline{e\;}{\;}_{bck}\right)\\ -\frac{1}{\omega^{3}}M_{S}Im\left({\tilde{R}}_{b}^{-1}\underline{e\;}{\;}_{bck}\right) & -\frac{1}{\omega^{2}}M_{S}Re\left({\tilde{R}}_{b}^{-1}\underline{e\;}{\;}_{bck}\right) \end{array}\right]\left[\begin{array}{c} \omega_{p}^{2}\\ \omega_{p}^{2}\nu \end{array}\right].$$

Problem (14) can be solved as a whole or by splitting it into two subsequent subproblems wherein the first step copes with the retrieval of the plasma frequency (i.e., the real part of the plasma contrast) and the second one dealing with the retrieval of the collision rate which is related to the imaginary part of the plasma contrast. This approach has been proposed in [7] wherein, considering the very low effect of the plasma losses on scattered field, the overall problem can be “decoupled”. This notwithstanding, in the following, we concentrate our attention only on the retrieval of the plasma angular frequency. As a matter of fact, experimental studies state that the collision rate is almost one order of magnitude smaller that the cyclotron frequency, which in turn, is much lower that the probing frequency. This entails to cope with a lossless plasma and the whole entire problem (14) will be reduced to the following one:

(15) $$\left[\begin{array}{c} Re(\underline{f\;}{\;}_{sct})\\ Im(\underline{f\;}{\;}_{sct}) \end{array}\right]=\left[\begin{array}{c} -\omega_{max}^{2}\frac{\omega }{\omega^{2}}M_{S}Re\left({\tilde{R}}_{b}^{-1}\underline{e\;}{\;}_{bck}\right)\\ -\omega_{max}^{2}\frac{\omega }{\omega^{3}}M_{S}Im\left({\tilde{R}}_{b}^{-1}\underline{e\;}{\;}_{bck}\right) \end{array}\right]\left[\begin{array}{c} \frac{\omega_{p}^{2}}{\omega_{max}^{2}} \end{array}\right],$$

with $\omega_{max}^{2}$ the maximum probing angular frequency used as normalization constant introduced for numerical stability of the inversion process [7].

### 3.2. Sparsity-Promoting Recovery Approaches

Equation (15) is a linear but still ill-posed one which must be solved in a regularized fashion. However, as stated in [10] and investigated in the case of plasma [7], minimum energy solutions approaches, such as Tikonov regularization [21,22] are completely unable to recover neither the shape nor the constitutive parameters of arbitrarily shaped plasma slabs. For this reason, CS-based approaches are worth to be considered, since they are in principle suitable to overcome the intrinsic limitations of the minimum energy solution related to the kind and number of actual parameters which can be conveyed back from multifrequency single view inverse scattering problem.

Let us consider a generic linear problem of the following kind:

(16) Φx=f,

*x* is the N×1 unknown vector and Φ is the M×N matrix, the so-called sensing matrix, and *f* the M×1 data vector of the multifrequency scattered field ordered according to (15). Let us suppose that a convenient representation matrix Ψ exists such that the unknown can be expanded as x=Ψs with only a small number of the coefficients *s* different from zero. According to CS theory, it is possible to solve the inverse problem (16) even if M<<N but is sufficiently larger than the number *S* of coefficients different from zero (with S<M<N). However, it is worth underlining that the number of the measurements *M* should be anyway in the order of the degrees of freedom of the scattered field [7], which in the case of the slab can be calculated as [10]:

(17) $$I=2L_{slab}\sqrt{\epsilon_{b}}\left(\frac{\lambda_{M}}{c_{0}}-\frac{\lambda_{m}}{c_{0}}\right),$$

$L_{slab}$ being the extent of the inhomogeneous slab, and $\lambda_{m}$ and $\lambda_{M}$ the minimum and maximum wavelength used to probe the scenario. Accordingly, it is possible to solve the inverse linear problem ΦΨs=Ps=f by means of different optimization constrained problems [23,24] based on the minimization of the $\ell_{1}$-norm of the unknown when represented in a proper sparse basis. The commonly adopted one in imaging problems, is the rectangular pulse basis function, commonly known as pixel and voxel in 2D and 3D, respectively. However, adopting such a basis, sparse recovery approaches are successful only if the scatterer resembles a point-like scatterer, whereas, in the case at hand, the plasma embeds almost the whole chamber which, in turn, is several probing wavelengths extended.

On the other hand, if a step-wise constant representation of the unknown profile is adopted, which is a reasonable assumption for the problem at hand, $\ell_{1}$ optimization can be still exploited. One possibility is offered CS-based approaches involving as objective function the first derivative of the unknown [7]. For these reasons, we could consider the following recovery problems:

(18) $$\begin{array}{ccc} \mathrm{argmin}\;{∥\frac{d({\tilde{\omega }}_{p}^{2})}{dz}∥}_{\ell_{1}} & \mathrm{subject}\;\mathrm{to} & {∥P{\tilde{\omega }}_{p}^{2}-f∥}_{2}\leq \delta ,\;\;\;{\tilde{\omega }}_{p}^{2}\geq 0, \end{array}$$

wherein ${\tilde{\omega }}_{p}^{2}=\frac{\omega_{p}^{2}}{\omega_{max}^{2}}$. In (18), ${∥\cdot ∥}_{\ell_{1}}$ and ${∥\cdot ∥}_{2}$ denote the $\ell_{1}$- and $L^{2}$-norms, respectively, and the parameter δ, which depends on the measurement error (noise on data) and model error (field approximation), is a positive defined parameter. In (18) the minimization of the $\ell_{1}$-norm promotes the search of solutions with a sparse derivative among all the solutions consistent with the measured data (within the given error threshold δ). In addition, taking into account the real positive value of the unknowns, a further constraint (${\tilde{\omega }}_{p}^{2}\geq 0$) can be enforced in (18) in order to reduce the search space by convex optimization and improve the reconstruction results. The approach (18) is known as basis pursuit denoising (BPDN) or least absolute shrinkage and selection operator (LASSO) problem [24].

In [7], the sparsity-promoting approaches (18) have been proved to be capable in retrieving synthetic step-wise constant axial plasma profile which can be represented with few coefficients in terms of their first derivative. However, as the 1D single view multifrequency inverse scattering problem is severe limited, by its very nature, in terms of number of degrees of freedom (*I*), the number of non-null coefficient of any suitable representation basis should stay very few in order to achieve reliable reconstructions. This means that when smoother ad smoother profiles are considered, the adopted sparsity-promoting approach (18) is expected to be unsatisfactory.

For this reason, a “relaxed” version of (18) can be considered, i.e.,:

(19) $$\begin{array}{ccc} \mathrm{argmin}\;{∥P{\tilde{\omega }}_{p}^{2}-f∥}_{2} & \mathrm{subject}\;\mathrm{to} & {∥\frac{d({\tilde{\omega }}_{p}^{2})}{dz}∥}_{\ell_{1}}\leq \gamma ,\;\;\;{\tilde{\omega }}_{p}^{2}\geq 0, \end{array}$$

wherein the role of the objective function with that of the constraints are exchanged. Approach (19) is expected to work better than (18) with profiles which are not exactly step-wise constant. Also, in this case, the assessment of the parameter γ is not a simple task. In this respect, prior information can be exploited to assess the optimal choice of the regularization parameter that mainly depends on the sparsity of the unknown at hand. However, the optimal choice of this threshold cannot be a priori established, pointing out one could a priori establish the range of the threshold and compare the results obtained for different values of γ.

## 4. Numerical Assessment Towards Benchmark Examples

The two benchmarks we refer to as “flat-top” and “hollow-core”, respectively, represent expected electron density axial distribution in ECRIS-like devices [5]. The synthetic data have been generated considering the convolution between a rectangular window and a Hann window large 10 cm and 0.7 cm, respectively with a discretization grid of 256 cells. As a result, smoother profiles than those tackled in [22] can be considered. The total and the background fields have been simulated in the frequency range [17.5–34.5] GHz considering 87 evenly spaced frequency values in the solution of the forward problem stated by (3), thus the scattered field data has been obtained as difference between them. The solution of the forward problem can be performed by means of a standard conjugate gradient scheme as that proposed in [16]. The choice of the adopted frequency band is related to several requirements. The first one is the need to consider magnetically confined plasma as isotropic and weak scattering medium, and such assumption is fulfilled when the probing frequency is much higher than the cyclotron frequency, hereafter considered at 2.45 GHz. The second one, is related to the need for working with a large bandwidth in order to achieve the number of degrees of freedom *I* as large as possible, see Equation (17). Last, but not least, at this frequency band, also in centimeter-sized compact reactors, the propagation can be reasonably approximated as one-dimensional one along the chamber’s axis. According to the above reasonings, we have set the frequency band of the fundamental mode in a rectangular waveguide WR34 that can be used to feed a horn antennas opening in the front and back walls of the plasma reactor [3].

We have considered three kind of measurement/background configurations which resemble possible experimental setup:

1. A single transmitting and receiving antenna measuring the reflection coefficient in a free-space homogeneous background (reflection-only measurement);
2. Single transmitting and receiving antenna measuring the reflection coefficient in presence of a PEC surface;
3. A transmitting and receiving antenna measuring the reflection coefficient and a receiving antenna measuring the transmission coefficient in a homogeneous free-space background (reflection and transmission measurement).

The three cases above can be seen a possible operational scenario where the reflection from metallic environments introduced by the plasma reactor can be neglected or not. This is the case, for example, when large and small reactors are in order, respectively.

To simulate the above measurement/background configurations, an absorbing, or free-space condition, can be very easily introduced, in the case at hand, by considering losses in PML layers ($z_{1}-d\leq z\leq z_{1}$ and $z_{2}\leq z\leq z_{2}+d$) where the medium is gradually modified introducing losses according appropriate permittivity and permeability $\epsilon_{r}\left[1-j\sigma \left(z\right)\right]$, $\mu_{r}\left[1-j\sigma \left(z\right)\right]$ to have ideally no reflections from the PML [11,12,14].

For the sake of simplicity, we have chosen a simple polynomial grading for the PML layers conductivity profiles: $\sigma \left(z\right)=\sigma_{max}{\left(\frac{z_{1}-z}{d}\right)}^{m}$ for $z_{1}-d\leq z\leq z_{1}$ and $\sigma \left(z\right)=\sigma_{max}{\left(\frac{z-z_{2}}{d}\right)}^{m}$ for $z_{2}\leq z\leq z_{2}+d$; but more complex profiles can be used [25]. On the other hand, when PEC layer is considered, the PML is completely removed and perfect reflecting surface boundary conditions are enforced at $z=z_{2}$.

It is worth pointing out that for the solution of the forward problem, the plasma profiles are assumed to be lossy with the collision rate profile one order of magnitude smaller than the plasma angular frequency. As a result, this introduces a model error in the scattered field data which avoids the so-called “inverse crime”. Furthermore, the scattered field data have been corrupted by random gaussian white noise of 5%. Finally, the number of the cells in the computational grid are set to N=256.

Problem (19) has been solved by means of the environment CVXPY 1.0 for the solution of convex optimization problems [26]. In doing this, the choice of the threshold value γ has been set equal to the exact $\ell_{1}$-norm of the actual unknown. Although this exact value cannot be chosen in any actual experiment, it allows an understanding of how the value of γ can be set on the base of expected a priori information on the degree of sparsity of the profile. Furthermore, for our investigation, such a choice allows the making of a fair comparison among the achieved results in the three different cases. For the two benchmarks profiles at hand it is γ=6.22 (flat-top) and γ=7.14 (hollow-core)

Finally, the required time to solve the inverse scattering problems takes very negligible time (below few seconds on a standard PC) due to the one-dimensional nature of the problem and then the size of the relevant matrix operator.

### 4.1. Reflection-Only Measurement with Single Antenna

We have considered free-space background with PML layers with thickness of 2 cm, σ=6, m=4, for a sketch the reader can refer to Figure 1a without considering the rightmost antenna. On the other hand, we have considered a non-homogeneous background with a PEC boundary condition on the right hand side of the computational domain ($z_{2}=25$ cm) to simulate the plasma chamber with only one antenna, see Figure 1b.

The imaging domain, $\left[z_{min},z_{max}\right]$, is long 20 cm with the source point $z^{\left(\mathrm{View}\right)}$ and the measurement point $z^{\left(\mathrm{Meas}\right)}$ placed at 2.5 cm far from the imaging domain both in the case of single antenna and two antennas.

**Figure 2:**
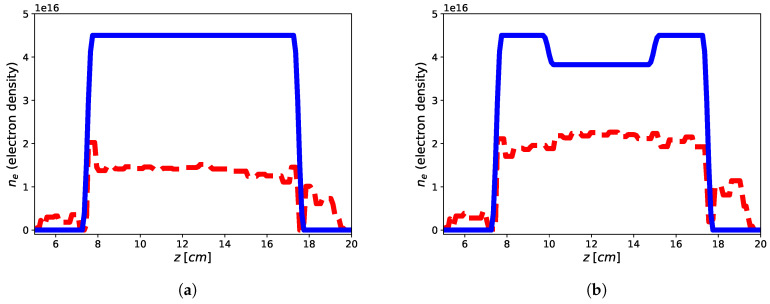
Reconstruction of the flat-top (**a**) and hollow core (**b**) plasma profiles using one tx-rx antenna in homogeneous vacuum background by means of approach (19). Blue continuous line: electronic density profile, red dashed line: retrieved electronic density profile.

**Figure 3:**
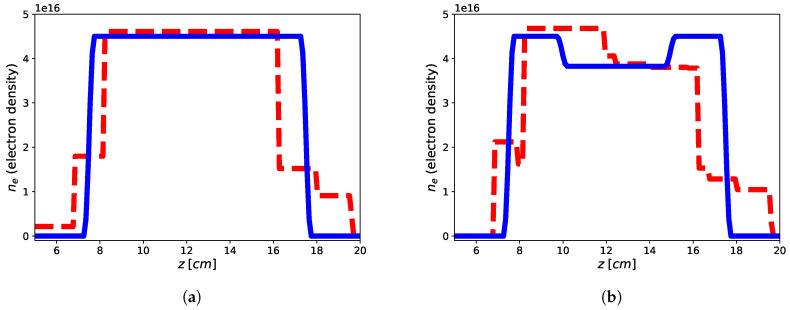
Reconstruction of the flat-top (**a**) and hollow core (**b**) plasma profiles using single tx-rx antenna in non-homogeneous background with a PEC reflecting surface by means of approach (19). Blue continuous line: electronic density profile, red dashed line: retrieved electronic density profile.

The imaging results are reported in Figure 2 and Figure 3 for both the homogeneous and non-homogeneous background, respectively. As it can be see, in the case of PEC layer, the reconstructions brings better result with a reconstruction error (defined as $err=\frac{{∥x_{true}-x_{rec}∥}_{2}^{2}}{{∥x_{true}∥}_{2}^{2}}$ ) of 9.39% for the flat-top and 13.03% for the hollow core, with the plasma profile not heavily underestimated as in the case of the homogeneous background, wherein the reconstruction errors achieve 47.85% for the flat-top and 27.02% for the hollow core. This means that the presence of a reflecting surface allows to the achievement of better results in terms of accuracy of the reconstructed profiles. On the other hand, it is worth underlining that while the reconstruction of the flat-top profile is almost satisfactory, Figure 3a, in the case of the hollow-core profile, neither the presence of the PEC allows imaging of the main feature, although differences about the two different recovered profiles are evident, see Figure 3a,b. This may be due to a lower degree of sparsity of the hollow-core profile than that of the flat-top one and to the requirement by (19) to find solutions (profiles) with bounded $\ell_{1}$ norm compatible with the objective function.

### 4.2. Reflection-Transmission Measurement with Two Antennas

In this case, two antennas are considered, see Figure 1a, with one (active) antenna radiating the probing field and measuring the reflection coefficient, and a second (passive) antenna measuring only the transmitting coefficient. Opposite to the case with a single antenna, in this case the non-homogeneous background accounting for two PEC surfaces has not been considered. First, from a modeling point of view, the use of PEC surfaces entails an electromagnetic cavity which enforces solutions only at the resonance frequencies given by $f_{n}=\frac{nc_{0}}{2L_{\mathrm{cavity}}}$, i.e., when the cavity length ($L_{\mathrm{cavity}}$) is an integer multiple of the probing half-wavelength, for a given finite set of integer $n^{*}$ indexes depending on the adopted frequency band. Secondly, when the plasma partially embeds the cavity, the resonance frequencies shift in frequency, so that the expected large error on the background field approximation prevent using a linear model such as the Rytov approximation. Finally, from an applicative point of view, since in general, the front and back walls of the plasma reactors host two aperture antennas [3], just a (possible small) fraction of the transmitted microwave power will be reflected, while the most part will be gathered by the antennas. For these reasons, we have considered only homogeneous background.

**Figure 4:**
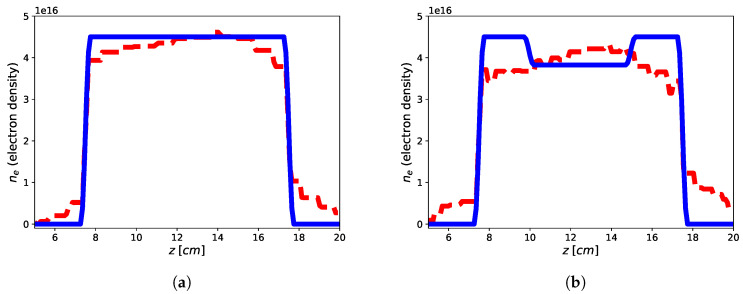
Reconstruction of the flat-top (**a**) and hollow core (**b**) plasma profiles using two antennas in homogeneous free-space background by means of approach 19. Blue continuous line: electronic density profile; red dashed line: retrieved electronic density profile.

The imaging results are reported in Figure 4 and the reconstruction errors achieve err=1.01% and err=3.56% for the flat-top and hollow core, respectively. Accordingly, it can be shown as the presence of a second passive antenna achieve better results in terms of accuracy of the retrieved profiles when compared to the single antenna in free-space homogeneous background. On the other hand, while the reconstruction of the flat-top profile is nearly optimal, also in this case, the reconstruction of the hollow-core profile is not satisfactory to apprise the main feature of the profile, although some small differences between the two retrieved profile are evident (as in the case of single antenna and PEC surface) and the same possible explanations hold true.

## 5. Discussion of the Results through Singular Value Decomposition Analysis

To understand the different results achieved under the two different considered measurement configurations and related backgrounds, we have considered the SVD analysis of the relevant scattering operator P for the different analyzed cases.

First of all, it is worth underlining that even if we are not considering minimum energy solution approaches ($L^{2}$-norm), the SVD analysis can be retained still valid to understand the main features among the different measurement configurations and backgrounds, since we are considering sparse promoting approaches rather than exact compressive sensing-based approaches. Indeed, (19) entails a standard minimum energy solution objective function equipped with a regularization (penalty) $\ell_{1}$-norm enforcing step-wise sparsity. For this reason, we numerically perform the SVD of the relevant scattering operator with the aim to give a qualitative interpretation of the different results shown in the previous Section.

Let us denote with $\{u_{n}$, $\sigma_{n}$, $v_{n}\}$ the singular value decomposition of the matrix operator P mapping the data-to-unknown relationship in (19), wherein $u_{n}$ represents the left singular vectors (spanning the object space), $v_{n}$ the right singular vectors (spanning the data space) and $\sigma_{n}$ the singular values, in such way $Pu_{n}=\sigma_{n}v_{n}$ and $P^{+}v_{n}=\sigma_{n}u_{n}$, wherein ${}^{+}$ stand for the adjoint of the operator [18].

For all the measurement configurations adopted in the numerical analysis, we have analyzed the following metrics based on the SVD analysis, i.e.,:

1. *behavior of the singular values* —the logarithmic plot of the singular values as ordered in non-increasing fashion. Indeed, as the scattering operator is a compact one [18], its singular values exhibits an exponential decay after a given threshold index *I* (analytically expressed by (17)), which indicates the maximum number of the degrees of freedom, and hence of the parameters which can be conveyed back by the recovery procedure;
2. *spectral coverage* (SC) defined as:
  (20) $$SC=\sum_{n=0}^{N_{T}}{\left|{\tilde{U}}_{n}\right|}^{2}$$
  wherein $\tilde{U}$ is the Fourier Transform of the left singular vectors and $N_{T}$ the truncation index used as regularization parameter in TSVD approach [10]. It is a measure of the class of profiles which can be actually retrieved in the object domain by the inversion process [10].
3. *point spread function* (PSF) defined as [27]:
  (21) $$PSF\left(z-z_{0}\right)=\sum_{n=0}^{N_{T}}u_{n}\left(z\right)u_{n}{\left(z_{0}\right)}^{*}$$
  wherein ${\left(\right)}^{*}$ stands for conjugation, and $z_{0}$ the abscissa with respect the PSF is considered. For the case at hand, we set $z_{0}=12.5$ cm, which is the center of the imaging domain. The point spread function is a direct measure of the ultimate attainable spatial resolution [27].

**Figure 5:**
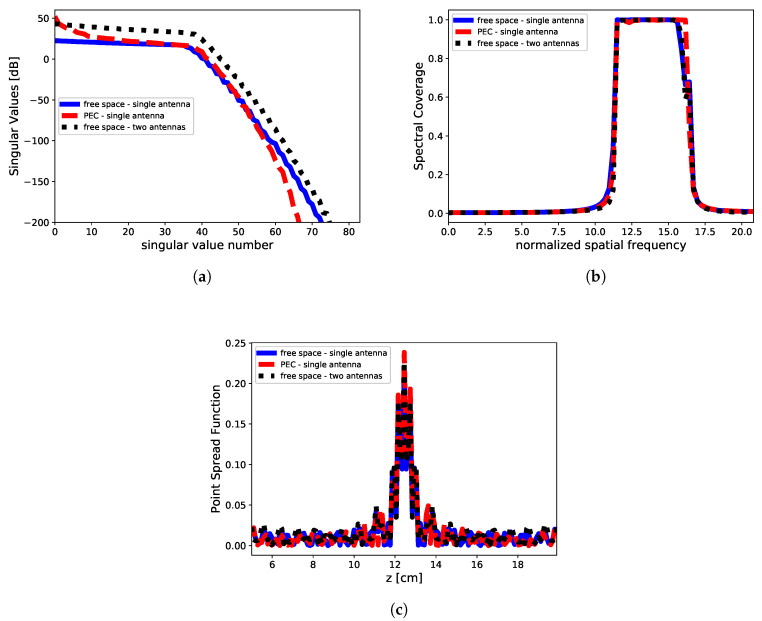
Singular value decomposition analysis. (**a**) Singular values, (**b**) spectral coverage and (**c**) point spread function in the case of single antenna free-space background (solid blue line), single antenna inhomogeneous PEC surface (red dashed line) and two antennas free-space background (dot black line). The cutoff value is $N_{T}=40$ according to the change of slope in the singular values behavior, while the spatial frequency in SC are normalized to the extent of the imaging domain.

The SVD analysis is reported in Figure 5. It can be seen that the singular values are different for the considered configurations. Indeed, for the PEC case the lower order singular values show a greater magnitude while vanishing to zero (like in the case of the free-space background configuration). On the other hand, in the case of two antennas in homogeneous background, the magnitude of the singular values keeps almost always greater than the single antenna (without and with PEC).

The SVD analysis give us the fundamental answer to understand the differences in the reconstruction process for the three adopted measurement configurations. Indeed, two main comments are in order. First, the presence of a perfect reflecting plane (PEC) introduces an increase in the singular values magnitude as compared to the homogeneous case. This is actually shown by the blue and red dashed line in Figure 5a. Interestingly, such an increase entails the advantage to deal with a more stable inverse scattering problem and with an increase of the information content [9]. Second, when we move to the case of two antennas, it can be easily understood as this configuration is almost equivalent to the case of single antenna operating in presence of a PEC surface. Indeed, this can be explained considering that the PEC surface acts as a “secondary” antenna which radiates (reflects) the field in such a way a virtual transmission coefficient can be measured by the actual antenna, as superimposed to the reflection coefficients that it would be measured without the PEC surface. As a result, when two antennas are considered, the reconstruction result achieves better results as in presence of a PEC surface. This analysis shows that the use of a second passive antenna is almost equivalent to have a reflecting surface behind the plasma slab. Anyhow, in all those cases wherein free-space approximation can be assumed (f.i., large meter-sized machine), two antennas can be used to achieve an enhancement in the reconstruction process. This has an immediate fallout in the design of the measurement setup depending on the constraints enforced by the experimental facilities.

On the other hand, the SVD analysis shows also that SC and PSF attained in three measurement configurations are identical, unless some very negligible differences due to numeric evaluation of the singular value system. These are confirmed by the same reconstruction performance achieved by the single antenna with PEC surface and two antennas in homogeneous background, for which the harmonic content is almost exactly the same. As a result, the improvement in the reconstruction in presence of a PEC surface can be attributed only to the difference in the singular values spectrum.

## 6. Conclusions

In this paper, we have addressed microwave imaging profilometry through the solution of an inverse scattering problem with a finite-difference formulation which takes into account, under the adopted assumptions, the effects of the metallic case represented by the plasma reactor. It has been shown that the presence of a metallic surface profoundly benefits the inversion recovery process when faced with sparsity-promoting approaches. In addition, the developed analysis shows the equivalence among two possible measurement configurations which are a single transmitting-receiving antenna in presence of a PEC surface and two antennas (one active and the second passive) able to measure also the transmission coefficients. Although the imaging capabilities are enhanced in presence of totally reflecting surface and with two antennas, microwave imaging profilometry still shows severe limitations in terms of accuracy in the recovery of arbitrary-shaped electron density profiles, i.e., not exactly step-wise constant profiles. Further activities are currently being devoted to exploit more efficient unknown representations and possible a priori information in order to achieve better reconstructions accuracy and to deal with a class of profiles wider than that considered in this paper.
